# A risk scoring system for predicting *Streptococcus suis* hearing loss: A 13-year retrospective cohort study

**DOI:** 10.1371/journal.pone.0228488

**Published:** 2020-02-04

**Authors:** Ajaree Rayanakorn, Wasan Katip, Bey Hing Goh, Peninnah Oberdorfer, Learn Han Lee

**Affiliations:** 1 Novel Bacteria and Drug Discovery Research Group (NBDD), Microbiome and Bioresource Research Strength, Jeffrey Cheah School of Medicine and Health Sciences, Monash University Malaysia, Bandar Sunway, Malaysia; 2 Department of Pharmaceutical Care, Faculty of Pharmacy, Chiang Mai University, Chiang Mai, Thailand; 3 Biofunctional Molecule Exploratory Research Group (BMEX), School of Pharmacy, Monash University Malaysia, Bandar Sunway, Malaysia; 4 College of Pharmaceutical Sciences, Zhejiang University, Hangzhou, China; 5 Health and Well-Being Cluster, Global Asia in the 21st Century (GA21) Platform, Monash University Malaysia, Bandar Sunway, Malaysia; 6 Division of Pediatric Infectious Diseases, Department of Pediatrics, Faculty of Medicine, Chiang Mai University, Chiang Mai, Thailand; Universidade do Estado do Rio de Janeiro, BRAZIL

## Abstract

**Background:**

*Streptococcus suis (S*.*suis)* is an emerging zoonosis disease with a high prevalence in Southeast Asia. There are over 1,500 cases reported globally in which majority of cases are from Thailand followed by Vietnam. The disease leads to meningitis in human with sensorineural hearing loss (SNHL) as the most common complication suffered by the patients. Early diagnosis and treatment is important to prevent severe neurological complication. In this study, we aim to develop an easy-to-use risk score to promote early diagnosis and detection of *S*.*suis* in patients who potentially develop hearing loss.

**Methods:**

Data from a retrospective review of 13-year *S*.*suis* patient records in a tertiary hospital in Chiang Mai, Northern, Thailand was obtained. Univariate and multivariate logistic regressions were employed to develop a predictive model. The clinical risk score was constructed from the coefficients of significant predictors. Area under the receiver operator characteristic curve (AuROC) was identified to verify the model discriminative performance. Bootstrap technique with 1000-fold bootstrapping was used for internal validation.

**Key Results:**

Among 133 patients, the incidence of hearing loss was 31.6% (n = 42). Significant predictors for *S*. *suis* hearing loss were meningitis, raw pork consumption, and vertigo. The predictive score ranged from 0–4 and correctly classified 81.95% patients as being at risk of *S*.*suis* hearing loss. The model showed good power of prediction (AuROC: 0.859; 95%CI 0.785–0.933) and calibration (AuROC: 0.860; 95%CI 0.716–0.953).

**Conclusions:**

To our best knowledge, this is the first risk scoring system development for *S*.*suis* hearing loss. We identified meningitis, raw pork consumption and vertigo as the main risk factors of *S*.*suis* hearing loss. Future studies are needed to optimize the developed scoring system and investigate its external validity before recommendation for use in clinical practice.

## Introduction

*Streptococcus suis (S*.*suis)* is a gram-positive alpha-hemolytic bacterium mainly in pigs which can causes serious infection in human with a high prevalence in Southeast Asia. There were over 1,500 *S*.*suis* cases reported worldwide, with the highest incidence in Thailand (> 600 cases) [1, 2]. About two thirds of *S*.*suis* infected patients would develop meningitis in which sensorineural hearing loss (SNHL) is the most common complication among most survivors [2]. SNHL is usually irreversible despite adequate treatment upon the sign of hearing loss occurs. Majority of patients experience persistent hearing loss upon acquiring the infection which is mainly bilateral [3, 4]. This may cause physical and emotional distress as well as constraint the economics of a nation.

*Streptococcus suis* SNHL is poorly diagnosed because patients do not undergo audiometry screening until they complaint of severe hearing disorder. By then, the prognosis of the disease is severe with slim percentage of recovery. Prior animal experiments and electrophysiological studies in human revealed that the underlying mechanism of hearing loss is probably from bacterial invasion through cochlear aqueduct, which causes suppurative labyrinthitis [5, 6]. This inflammatory disorder in the inner ear has been identified as the responsible site of deafness and meningitis auditory lesion [6, 7]. SNHL was found to evolve at the early stage of meningitis and could progress to permanent hearing loss if meningitis had not been promptly treated appropriately [6]. Therefore, early diagnosis and immediate treatment are essential to reduce detrimental consequences mainly SNHL from *S*.*suis* infection. This study aims to develop an easy-to-use risk score to promote early diagnosis and detection of *S*.*suis* infected patients who are prone to hearing loss in primary settings.

## Methods

### Study design and setting

The data was collected as a part of a retrospective cohort study over 13-year period at Chiang Mai University Hospital (CMUH) [8], a 1400-bed tertiary teaching hospital. The hospital is the largest in northern, Thailand and the fourth largest hospital in the country where most of patients in northern region from 17 provinces are referred to for tertiary care (see S1 Table). Cultural eating habit of raw pork dishes and fermented raw pork is commonly practiced in northern, Thailand. This deep-rooted cultural eating behavior is a major route of the disease transmission and contributing factor of a high prevalence of the disease in this region.

### Study population and data collection

**Fig 1:**
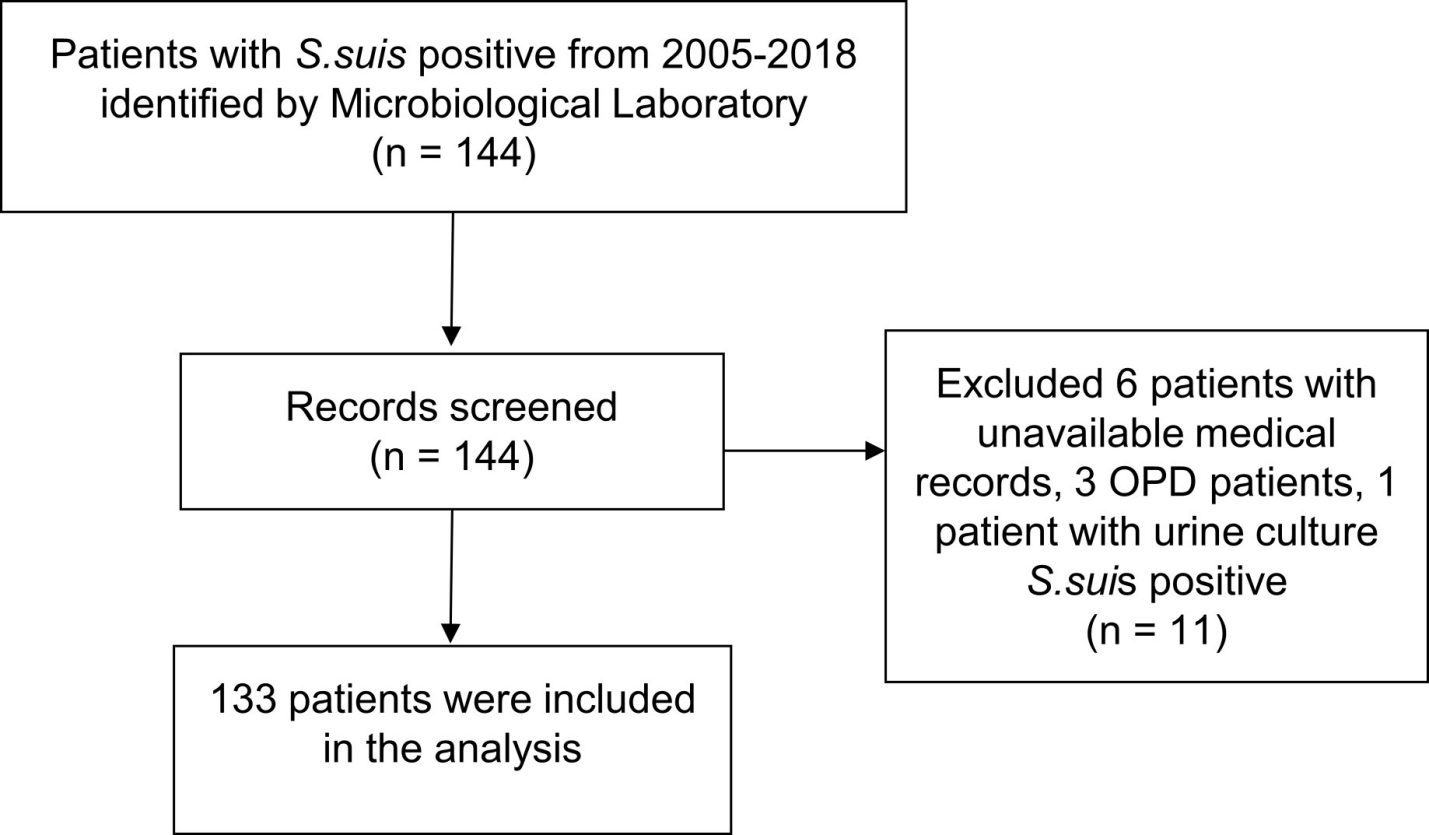
Patients identification and selection.

*S*. *suis* positive cases were identified based on the microbiology laboratory data available and hospital numbers (HNs) admitted from May 2005 to December 2018. *S*. *suis* cases were confirmed by blood or cerebrospinal fluid (CSF). All confirmed *S*.*suis* cases with available medical records were included in this analysis (Fig 1) [8].

The data collected included patient demographics, clinical characteristics and manifestations, outcomes and treatments. Patient data were collected and managed using REDCap electronic data capture tools hosted at Research Electronic Data Capture, Monash University Malaysia, a secure, web-based application designed to support data capture for research studies [9]. Patient information collected was anonymous and treated as confidential throughout the process of data collection and management.

### Outcomes

*S*. *suis* meningitis was confirmed by cerebrospinal fluid (CSF) culture with compatible clinical presentation. An audiogram was used to diagnose and monitor the degree of hearing ability. The degree of hearing loss was assessed by otorhinolaryngologists based on individual patients’ hearing thresholds in decibel (dB) at different frequencies. The level of hearing loss was classified into mild, moderate, severe and profound. Presbycusis or any pre-existing hearing loss would be excluded. Endocarditis and site of vegetation was assessed by echocardiography.

### Ethical considerations

The study was approved by the Research Ethics Committee, Faculty of Medicine, Chiang Mai University (IRB no.010/2018) and Monash University Human Research Ethics Committee (MUHREC) (Project no.12225). Informed consent forms were not required in this retrospective review. The study reporting was in accordance to the Transparent Reporting of a Multivariable Prediction Model for Individual Prognosis or Diagnosis (TRIPOD) statement [10] (S1 TRIPOD Checklist).

### Statistical analysis

Demographics and clinical characteristics were initially analyzed descriptively. Variables are defined as risk factors associated with an increased risk of *S*.*suis* hearing loss. Univariate analysis was performed to compare potential predictors between hearing loss and non-hearing loss patients. For continuous variables, Student’s t test was used in case of normal distribution whereas Mann-Whitney-Wilcoxon test was employed if the data is not normally distributed. Chi-square test or Fisher’s exact test was used for categorical variables. We performed both complete case analysis and multiple imputation using predictive mean matching with 20 iterations for missing Glasgow Coma Scale (GCS) values [11]. Variables with p-value less or equal to 0.10 would be carried forward in multivariate analysis. Potential collinearity was also explored before building the predictive model. Forward step-wise logistic regression was utilized to identify significant predictors for *S*.*suis* hearing loss. Predictors that remained significant at p-value ≤ 0.10 would be included in the final parsimonious model.

### Risk score development and internal validation

A clinical scoring system was developed by transforming regression coefficients into item scores. Each item score was generated by dividing with the smallest coefficient in the model and rounding the number to the closest integer [12]. The model discrimination was determined from the area under the receiver-operator characteristic curve (AuROC) or the concordance (C) statistics. An area under the curve (AUC) of 1.0 reflects a perfect discrimination performance whereas an AUC of 0.5 implies a completely no discriminative ability [13]. Calibration of the final model was tested using Hosmer-Lemeshow good-ness-of-fit test and the calibration plot portraying predicted vs. observed probability of *S*.*suis* hearing loss was illustrated. The risk stratification was done according to scores distribution into four quartile groups. The classification performances including sensitivity, specificity, positive (LR+) and negative likelihood ratios (LR-) were also calculated. A bootstrap with 1,000 replications technique was used for internal validation to correct for optimism [14]. All statistical analyses were done by STATA 14.2 (College Station, Texas, USA).

## Results

One hundred and thirty-three patients with *S*.*suis* infection were included in this analysis, majority were males (67.2%). Table 1 summarizes the patient demographics and medical history. More than one-third of patients had a history of raw pork consumption and nearly half of the patients were regular alcohol drinkers. Valvular heart disease was the most common underlying disease followed by Diabetes Mellitus (DM) and spondylodiscites. A total of 42 patients (31.58%) experienced SNHL from *S*.*suis* infection in which 13 (9.77%) were mild SNHL, 7 (5.26%) were moderate SNHL, 4 (3.01%) were severe SNHL and 12 (9.02%) were profound SNHL. All SNHL still persisted based on audiometry upon discharge and latest follow-up visit up to December 2018.

**Table 1:** Patient characteristics.

Characteristics	Total (n = 133)
**Age (year) (mean±SD)**	56.47± 13.68
**Male**	92 (69.17%)
**GCS**	12.65± 3.15
**Risk behaviors**	
** • Consumption of raw pork**	49 (36.84%)
** • Recent contact with pigs/pork exposure**	5 (3.76%)
** • Pig related occupation**	3 (2.26%)
** • Skin injury**	2 (1.50%)
** • Alcohol drinking**	66 (49.62%)
** • No**	24 (18.05%)
**Underlying diseases**	
** • DM**	26 (19.55%)
** • ALD**	16 (12.03%)
** • Splenectomy**	2 (1.50%)
** • Valvular heart disease**	44 (33.08%)
** • Cancer**	2 (1.50%)
** • Corticosteroid use**	3 (2.26%)
** • HIV/AIDS**	2 (1.50%)
** • Spondylodiscites**	27 (20.30%)
** • SLE**	21 (15.79%)
Exposure to onset (days) † (mean±SD)	7.67± 11.66
Time from exposure to admission (days) (mean±SD) ‡	13.55± 19.21

GCS, Glasgow coma scale; DM, Diabetes Mellitus; ALD, Alcoholic liver disease; HIV, Human immunodeficiency virus infection; AIDS, Acquired immune deficiency syndrome; SLE, Systemic lupus erythematosus

*Note*

†Available data from 37 patients

‡ Available data from 40 patients

Univariate analysis suggested association between *S*.*suis* hearing loss with raw pork consumption (p<0.001), valvular heart disease (VHD) (p = 0.009), alcoholic liver disease (ALD) (p = 0.093), acute meningitis (p<0.001), neck stiffness (p<0.001), infective endocarditis (IE) (p = 0.005), vomiting (p = 0.019), vertigo (p = 0.011) and lower level of serum creatinine (p = 0.018) and potassium (p = 0.002) (Table 2). Continuous variables were categorized into two groups to simplify application. For creatinine, the cut point was 2.0 mg/dL which was around 1.5 to 2 times compared to the mean value among *S*.*suis* hearing loss cases whereas 4.0 mmol/L was the cut-off point for serum potassium. GCS variable remained a non-significant predictor in both complete case analysis and multiple imputation data (S2 Table).

**Table 2:** Clinical characteristics of *S*. *suis* infected patients for hearing loss.

Characteristics	Hearing loss (n = 42)	Non-hearing loss (n = 91)	p-value
	N (%)	N (%)	
**Demographics:**			
**• Age (year) (mean±SD)**	54.62±12.12	57.33±14.33	0.290
** • Male**	29 (69.05)	63 (69.23)	0.568
**• Raw pork consumption**	25 (59.05)	24 (26.37)	<0.001
** • Alcohol drinking**	25 (59.52)	41 (45.05)	0.138
**Baseline characteristics:**			
**GCS †**	13.09±2.28	12.42± 3.52	0.318
**Microbiological results:**			
** • Time to microbiological cure**	9.86±11.07	8.33±15.29	0.526
• **Mean MIC to penicillin (μg/mL) ‡**	0.14±0.18	0.17±0.15	0.106
• **Mean MIC to ceftriaxone (μg/mL) ‡‡**	0.14±0.10	0.29±0.31	0.388
**Underlying disease**			
** • Valvular heart disease**	7 (16.67)	37 (40.66)	0.009
**• ALD**	2 (4.76)	14 (15.38)	0.093
** • DM**	5 (11.90)	21 (23.08)	0.162
** • Spondylodiscites**	6 (14.29)	21 (23.08)	0.354
**Major clinical manifestations**			
**• Acute meningitis**	34 (80.95)	16 (17.58)	<0.001
** • Neck stiffness**	31 (73.81)	16 (17.58)	<0.001
**• Septicaemia**	23 (54.76)	51 (56.04)	1.000
** • IE**	4 (9.52)	30 (32.97)	0.005
**• Vomiting**	14 (33.33)	13 (14.29)	0.019
** • Vertigo**	7 (16.67)	3 (3.30)	0.011
**Receiving steroids**	22 (53.66)	1 (1.10)	0.600
**Laboratory findings:**			
**• CSF protein††**	277.12±220.25	341.39±256.93	0.419
**• CSF glucose‡‡**	31.69±9.70	30.72±21.55	0.173
**• Creatinine (mg/dl)**	1.18±0.75	2.09±2.94	0.018
** • Potassium (mmol/L)±**	3.52±0.43	3.93±0.77	0.002

ALD, Alcoholic liver disease; CSF, Cerebrospinal fluid; DM, Diabetes Mellitus; GCS, Glasgow coma scale; IE, Infective endocarditis; MIC, Minimal Inhibitory Concentration

† Data available in 101 patients

‡ Data available in 57 patients

‡‡ Data available in 53 patients

†† Data available in 52 patients

± Data available in 127 patients

**Fig 2:**
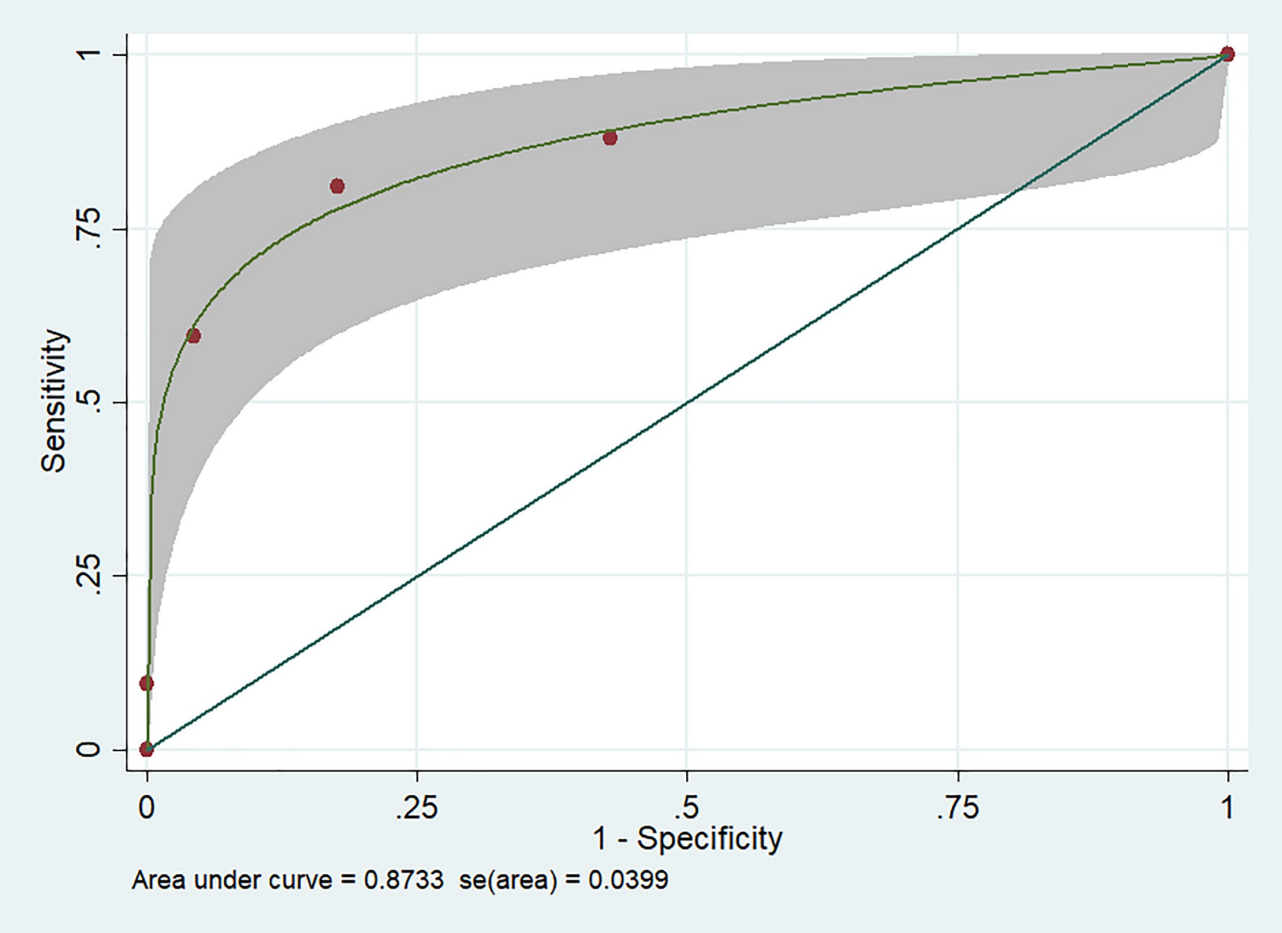
Area under the receiver-operator characteristic curve (AuROC) for the scoring system to predict *S*.*suis* hearing loss with 95% confidence interval.

Two predictors were removed after checking on collinearity which were neck stiffness and VHD whereas eight predictors (raw pork consumption, ALD, acute meningitis, IE, vomiting, vertigo, a low level of serum creatinine and potassium) were included in the stepwise forward logistic regression. There were three predictors remained in the model at p-value ≤ 0.1 which were meningitis, raw pork consumption and vertigo. The final parsimonious model at significant level ≤ 0.1 which provided optimal the area under the receiver operating characteristic curve (AuROC) was selected. The scoring system indicated a good performance in *S*.*suis* hearing loss prediction (AuROC 0.87; 95% CI 0.80–0.95) (Fig 2).

The C statistics or AuROC was 0.86 (95% CI 0.78–0.93) suggesting acceptable discrimination from *S*.*suis* hearing loss from non-hearing loss. The calibration plot and Hosmer-Lemeshow goodness-of-fit test indicated good model calibration (chi-square = 3.13, p-value 0.372), see S1 Fig and S3 Table.

The item scores were generated by dividing significant coefficients with the smallest coefficients. The clinical predictive score ranged from 0–4. The risk of *S*. *suis* hearing loss increases as the score increases (Table 3). The score cut-off point was identified based on the score distribution into four quartile groups which reflects LR+ of 1, 2, 3.9 and 13.5. Patients were classified into four groups according to the risk of low, moderate, high and very high risk. The score cut-off point at 3 out of 4 produced the optimum sensitivity and specificity with a sensitivity of 80.95% and 79.12% respectively. The classification, sensitivity, specificity, LR+ and LR- were presented in Table 4. Overall, 81.95% patients were correctly identified as being at risk or not being at risk of *S*.*suis* hearing loss.

**Table 3:** Derived item scores from multivariable logistic regression (n = 133).

Predictors	OR^a^	95%CI	p-Value	ROC Area (95%CI)	Βeta coefficient (intercept = -53.11)	Score
**Meningitis**						
** No**	1.00	reference	-		-	0
** Yes**	16.64	6.25–44.30	<0.001	0.82 (0.75–0.89)	2.812	2
**Raw pork consumption**						
** No**	1.00	reference	-		-	0
** Yes**	3.52	1.32–9.42	0.012	0.67 (0.58–0.75)	1.26	1
**Vertigo**						
** No**	1.00	reference	-		-	0
** Yes**	5.06	0.74–34.41	0.097	0.57 (0.51–0.63)	1.62	1

^a^ Model for Multivariable logistic regression using backward stepwise logistic regression

**Table 4:** Risk stratification of prediction values of *S*.*suis* hearing loss score.

Risk classification	Scores	Outcomes		Sensitivity (%)	Specificity (%)	Correctly classified (%)	LR+ (95%CI)	LR- (95%CI)
		Hearing loss	Non-hearing loss					
**Low**	1	18	39	100	0	31.58	1.00	0
**Moderate**	2	15	8	88.10	51.14	66.92	2.06	0.21
**High**	3	19	5	80.95	79.12	79.70	3.88	0.24
**Very high**	4	24	5	59.52	95.60	84.21	13.54	0.42

A bootstrap of 1000 sampling with replacement was performed for internal validation. The Somer’D coefficient which is an estimated correlation between the observed and predicted value of all boostrap data (D_boot_) and the original data (D_origin_) were 0.721 (95%CI 0.432–0.905) and 0.718 (95%CI 0.570–0.867) respectively. The average bias was -0.0022 (95%CI -0.002 to—0.002) indicating low bias and good calibration. The average C statistic from bootstraps was 0.860 (95%CI 0.716–0.953) with estimated bias of -0.001 (95%CI -0.001 to -0.001). The AuROC was 0.860 (95%CI 0.716–0.953).

## Discussion

To our best knowledge, this is the first risk scoring system development for *S*.*suis* hearing loss. We identified meningitis, raw pork consumption and vertigo as the main risk factors of *S*.*suis* hearing loss. The data was derived from the real setting in routine practice upon admission at Chiang Mai University Hospital (CMUH). Usually these data are available and do not require any invasive laboratory procedure. After evaluating its external validity, this simple scoring system might be useful to assess patients with *S*.*suis* hearing loss in hospital and primary care settings.

The mechanism of SNHL which is a remarkable consequence from acquiring *S*.*suis* infection in up to 50% of cases is assumed to be caused by the direct infection at the cochlea. The pathogen invasion through the cochlea aqueduct is believed to be from its exotoxin lytic action [7]. Presence of vertigo could be attributed from vestibular damage which may result in vestibular dysfunction. The benefit of steroids in reducing the likelihood of hearing loss has been established in pediatric meningitis but remained uncertain in adult population [15]. From our observation, audiometry test was generally performed upon patients’ compliant or presentation of hearing loss symptoms which may be too late to prevent the sequelae. Timely diagnosis and immediate antibiotic treatment before occurrence or sign of hearing loss symptoms are essential to prevent or reduce damage particularly SNHL caused by *S*.*suis* infection. This study is the first attempt to develop a risk score for predicting *S*.*suis* hearing loss as a tool to support clinicians in managing the disease and reduce the impact from its sequelae. The strength of the study is a reasonably large sample size of *S*.*suis* patients compared to previous studies [16, 17]. However, a number of limitations can be noted in our study. Missing data and recall bias may have arisen due to retrospective nature. Considering *S*.*suis* as a ‘rare’ disease, the data from over 13-year period was captured to get a high sample size. With this regards, the treatment and management may have changed over the years. However, audiometry was routinely used to diagnose SNHL at this setting throughout the whole study period and *S*.*suis* infection is still generally susceptible to common antibiotics such as penicillin and ceftriaxone. Therefore, this might not have substantially affected the results of the study. In addition, there were few predictors included in the model in which history of raw pork consumption may be potentially to be affected by a recall bias. As our main purpose was to develop a simple clinical prediction tool which is easy to be applied in primary setting, having these predictors (meningitis, raw pork consumption and vertigo) which are available upon patients’ presentation should be practical to assist with initial detection of potential hearing loss cases. Raw pork consumption was known to be a potential factor in acquiring the disease whereas documentation on medical history of such cases was limited. However, we still could identify more than one-third of patients with documented medical history of raw pork consumption whereas there were many fewer cases with this medical history could be confirmed in the previous retrospective study at the same setting [16]. An extensive medical record review to capture all relevant information may have contributed to this finding. Nevertheless, as traditional culture involving raw pork consumption is a well-known risk behavior among northern Thai population, doctors might have more likely asked patients about this risk behavior which might have been potentially subject to information bias. Therefore, generalizability of the finding should be done with caution. With limited study sample size at one tertiary hospital, the external validation of the model would not be possible. We contacted investigators who had previously conducted studies in *S*.*suis* patients for the purpose of external validation but we did not receive any response. According to the rule of thumb from a simulation study, the number of event per variable (EPV) of 10 or more was required to prevent bias in the regression coefficients [11]. In our study, there were 42 *S*.*suis* patients with hearing loss as convenience sample and there were three significant risk factors confirmed in the final model. Therefore, this should be considered to be sufficiently powered. Future studies to optimize such tool and investigation on its external validity should be mandated before implementation. However, robust statistical methods including the bootstrap of 1000 replications for internal validation were performed. The results demonstrated very close Somer’s D coefficients and very low bias between original and bootstrap data indicating good model calibration. The benefits of corticosteroids use in preventing SNHL from *S*.*suis* dexamethasone with antibiotics could not be determined in our study due to a relatively few number of patients received adjuvant corticosteroids with antibiotics. Apart from that most adjuvant corticosteroids were administered among those with moderate to profound SNHL after clinical features of hearing loss presentation. Finally, it should be noted that the participants included in the study were mainly from Northern, Thailand where traditional raw pork eating is practiced. This may limit generalizability in other settings especially where raw pork consumption is uncommon.

In conclusion, after external validation, our simple clinical risk score developed might be useful to aid clinicians in identifying patients who are likely to develop hearing loss from *S*.*suis* infection. Although this tool cannot replace clinical judgement, physicians can look upon these clinical characteristics in patients (meningitis, raw pork consumption and vertigo) for early detection of potential *S*.*suis* hearing loss cases and administrate immediate treatment to avoid long-term complications.

With an absence of vaccination for *S*.*suis* prevention, timely diagnosis and immediate antibiotic treatment before occurrence or sign of hearing loss symptoms are essential to prevent or reduce damage particularly SNHL caused by *S*.*suis* infection. Additionally, increased awareness among clinicians and microbiologists on *S*.*suis* infection as an emerging zoonosis is important to foster early disease detection, management, and prevention. Public awareness program and food safety campaign should also be considered for disease control and prevention.

## Supporting information

S1 TRIPOD Checklist Prediction Model Development [10]. (PDF)

S1 Checklist STROBE checklist. (PDF)

S1 Table List of Northern provinces of Thailand as of 2018. (DOCX)

S2 Table Clinical characteristics of *S*. *suis* infected patients for hearing loss based on imputed GCS. (DOCX)

S3 Table Hosmer-Lemeshow good-ness-of-fit test. (DOCX)

S1 Fig Calibration plot between predicted vs. observed probability of *S*. *suis* hearing loss. (TIF)

S2 Fig Discrimination of *S*. *suis* hearing loss based on *S*. *suis* hearing loss scores. Blue grey bars: non-hearing loss cases (N = 91); Red bars: hearing loss cases (N = 42). (TIF)
