# Supplementary material for: A risk scoring system for predicting Streptococcus suis hearing loss: A 13-year retrospective cohort study
Source: PLoS One. 2020 Feb 4;15(2):e0228488. doi: 10.1371/journal.pone.0228488 (PMC6999904; doi:10.1371/journal.pone.0228488)
Supplement: S1 Checklist — (PDF) [file pone.0228488.s002.pdf]

STROBE Statement—checklist of items that should be included in reports of observational studies

	Item No.	Recommendation	Page No.	Relevant text from manuscript
**Title and abstract**	1	(*a*) Indicate the study's design with a commonly used term in the title or the abstract	1; Title page,	"A risk scoring system for predicting *Streptococcus suis* hearing loss: A 13-Year Retrospective Cohort Study"
			3; Abstract	"Data from a retrospective review of 13-year *S.suis* patient records in a tertiary hospital in Chiang Mai, Northern, Thailand was obtained."
		(*b*) Provide in the abstract an informative and balanced summary of what was done and what was found	3; Abstract	"Methods<br>Data from a retrospective review of 13-year *S.suis* patient records in a tertiary hospital in Chiang Mai, Northern, Thailand was obtained. Univariate and multivariate logistic regressions were employed to develop a predictive model. The clinical risk score was constructed from the coefficients of significant predictors. Area under the receiver operator characteristic curve (AuROC) was identified to verify the model discriminative performance. Bootstrap technique with 1000-

				fold bootstrapping was used for internal validation.<br>Key Results<br>Among 133 patients, the incidence of hearing loss was 31.6% (n=42). Significant predictors for *S. suis* hearing loss were meningitis, raw pork consumption, and vertigo. The predictive score ranged from 0-4 and correctly classified 81.95% patients as being at risk of *S.suis* hearing loss. The model showed good power of prediction (AuROC: 0.859; 95%CI 0.785-0.933) and calibration (AuROC: 0.860; 95%CI 0.716-0.953)."
**Introduction**				
Background/rationale	2	Explain the scientific background and rationale for the investigation being reported	5; Introduction	"*Streptococcus suis* SNHL is poorly diagnosed because patients do not undergo audiometry screening until they complaint of severe hearing disorder. By then, the prognosis of the disease is severe with slim percentage of recovery."<br>"SNHL was found to evolve at the early stage of meningitis and could progress to permanent hearing loss if meningitis had

				not been promptly treated appropriately [6]. Therefore, early diagnosis and immediate treatment are essential to reduce detrimental consequences mainly SNHL from *S.suis* infection.”
Objectives	3	State specific objectives, including any prespecified hypotheses	5; Introduction	“This study aims to develop an easy-to-use risk score to promote early diagnosis and detection of *S.suis* infected patients who are prone to hearing loss in primary settings.”
**Methods**				
Study design	4	Present key elements of study design early in the paper	5: Methods, Study design and setting	“The data was collected as a part of a retrospective cohort study over 13-year period at Chiang Mai University Hospital (CMUH).”
Setting	5	Describe the setting, locations, and relevant dates, including periods of recruitment, exposure, follow-up, and data collection	5: Methods, Study design and setting	“The hospital is the largest in northern, Thailand and the fourth largest hospital in the country where most of patients in northern region from 17 provinces are referred to for tertiary care.”
			6; Methods, Study population and data collection	“*S. suis* positive cases were identified based on the microbiology laboratory data available and hospital numbers

				(HNs) admitted from May 2005 to December 2018". "The data collected included patient demographics, clinical characteristics and manifestations, outcomes and treatments."
Participants	6	(*a*) *Cohort study*—Give the eligibility criteria, and the sources and methods of selection of participants. Describe methods of follow-up *Case-control study*—Give the eligibility criteria, and the sources and methods of case ascertainment and control selection. Give the rationale for the choice of cases and controls *Cross-sectional study*—Give the eligibility criteria, and the sources and methods of selection of participants	6; Methods, Study population and data collection	"*S. suis* positive cases were identified based on the microbiology laboratory data available and hospital numbers (HNs) admitted from May 2005 to December 2018. *S. suis* cases were confirmed by blood or cerebrospinal fluid (CSF). All confirmed *S.suis* cases with available medical records were included in this analysis (Figure 1 Patient identification and selection) [8]."
		(*b*) *Cohort study*—For matched studies, give matching criteria and number of exposed and unexposed *Case-control study*—For matched studies, give matching criteria and the number of controls per case	N/A	
Variables	7	Clearly define all outcomes, exposures, predictors, potential confounders, and effect modifiers. Give diagnostic criteria, if applicable	6; Methods, Outcomes	"*S. suis* meningitis was confirmed by cerebrospinal fluid (CSF) culture with compatible clinical presentation. An audiogram was used to diagnose and monitor the degree of hearing ability. The degree

		of hearing loss was assessed by otorhinolaryngologists based on individual patients' hearing thresholds in decibel (dB) at different frequencies. The level of hearing loss was classified into mild, moderate, severe and profound. Presbycusis or any pre-existing hearing loss would be excluded. Endocarditis and site of vegetation was assessed by echocardiography."
	7; Methods, Statistical analysis	"Variables are defined as risk factors associated with an increased risk of *S.suis* hearing loss."<br><br>"Variables with p-value less or equal to 0.10 would be carried forward in multivariate analysis. Potential collinearity was also explored before building the predictive model. Forward step-wise logistic regression was utilized to identify significant predictors for *S.suis* hearing loss. Predictors that remained significant at p-value ≤ 0.10 would be included in the final parsimonious model."

Data sources/ measurement	8*	For each variable of interest, give sources of data and details of methods of assessment (measurement). Describe comparability of assessment methods if there is more than one group	6; Methods, Outcomes	"An audiogram was used to diagnose and monitor the degree of hearing ability. The degree of hearing loss was assessed by otorhinolaryngologists based on individual patients' hearing thresholds in decibel (dB) at different frequencies. The level of hearing loss was classified into mild, moderate, severe and profound. Presbycusis or any pre-existing hearing loss would be excluded".
			7, Methods, statistical analysis	"Univariate analysis was performed to compare potential predictors between hearing loss and non-hearing loss patients. For continuous variables, Student's t test was used in case of normal distribution whereas Mann-Whitney-Wilcoxon test was employed if the data is not normally distributed. Chi-square test or Fisher's exact test was used for categorical variables. We performed both complete case analysis and multiple imputation using predictive mean matching with 20 iterations for missing Glasgow Coma Scale (GCS) values [11]."

Bias	9	Describe any efforts to address potential sources of bias	7; Methods, Statistical analysis	"Potential collinearity was also explored before building the predictive model".
			8; Methods, Risk score development and internal validation	"A bootstrap with 1,000 replications technique was used for internal validation to correct for optimism [14]."
			11-12; Discussion	"An extensive medical record review to capture all relevant information may have contributed to this finding".
			12; Discussion	"We contacted investigators who had previously conducted studies in *S.suis* patients for the purpose of external validation but we did not receive any response."
Study size	10	Explain how the study size was arrived at	12; Discussion	"According to the rule of thumb from a simulation study, the number of event per variable (EPV) of 10 or more was required to prevent bias in the regression coefficients [11]. In our study, there were 42 *S.suis* patients with hearing loss as convenience sample and there were three significant risk factors confirmed in the final model."

Continued on next page

Quantitative variables	11	Explain how quantitative variables were handled in the analyses. If applicable, describe which groupings were chosen and why	9; Results	"Continuous variables were categorized into two groups to simplify application. For creatinine, the cut point was 2.0 mg/dL which was around 1.5 to 2 times compared to the mean value among *S.suis* hearing loss cases whereas 4.0 mmol/L was the cut-off point for serum potassium."
Statistical methods	12	(*a*) Describe all statistical methods, including those used to control for confounding	8-9; Results	"Univariate analysis suggested association between *S.suis* hearing loss with raw pork consumption ($p<0.001$), valvular heart disease (VHD) ($p=0.009$), alcoholic liver disease (ALD) ($p=0.093$), acute meningitis ($p<0.001$), neck stiffness ($p<0.001$), infective endocarditis (IE) ($p=0.005$), vomiting ($p=0.019$), vertigo ($p=0.011$) and lower level of serum creatinine ($p=0.018$) and potassium ($p=0.002$) (Table 2). Continuous variables were categorized into two groups to simplify application. For creatinine, the cut point was 2.0 mg/dL which was around 1.5 to 2 times compared to the mean value among *S.suis* hearing loss cases whereas 4.0 mmol/L was the cut-off point for serum potassium. GCS variable remained a non-

			significant predictor in both complete case analysis and multiple imputation data (Table S2 Clinical characteristics of *S. suis* infected patients for hearing loss based on imputed GCS).<br>Two predictors were removed after checking on collinearity which were neck stiffness and VHD whereas eight predictors (raw pork consumption, ALD, acute meningitis, IE, vomiting, vertigo, a low level of serum creatinine and potassium) were included in the stepwise forward logistic regression. There were three predictors remained in the model at p-value $\leq$ 0.1 which were meningitis, raw pork consumption and vertigo. The final parsimonious model at significant level $\leq$ 0.1 which provided optimal the area under the receiver operating characteristic curve (AuROC) was selected."
	(*b*) Describe any methods used to examine subgroups and interactions	N/A	
	(*c*) Explain how missing data were addressed	7; Methods, Statistical analysis	"We performed both complete case analysis and multiple imputation using predictive mean matching with 20 iterations for missing Glasgow Coma Scale (GCS) values [11]."

		(*d*) *Cohort study*—If applicable, explain how loss to follow-up was addressed<br>*Case-control study*—If applicable, explain how matching of cases and controls was addressed<br>*Cross-sectional study*—If applicable, describe analytical methods taking account of sampling strategy	9; Results	"GCS variable remained a non-significant predictor in both complete case analysis and multiple imputation data (Table S2 Clinical characteristics of S. suis infected patients for hearing loss based on imputed GCS)."
		(*e*) Describe any sensitivity analyses	N/A	
**Results**				
Participants	13*	(a) Report numbers of individuals at each stage of study—eg numbers potentially eligible, examined for eligibility, confirmed eligible, included in the study, completing follow-up, and analysed	6; Methods, Study population and data collection<br><br>8; Results	"*S. suis* positive cases were identified based on the microbiology laboratory data available and hospital numbers (HNs) admitted from May 2005 to December 2018. *S. suis* cases were confirmed by blood or cerebrospinal fluid (CSF). All confirmed *S.suis* cases with available medical records were included in this analysis (Figure 1 Patient identification and selection)."<br><br>"One hundred and thirty-three patients with *S.suis* infection were included in this analysis, majority were males (67.2%)."
		(b) Give reasons for non-participation at each stage	N/A	
		(c) Consider use of a flow diagram	N/A	

Descriptive data	14*	(a) Give characteristics of study participants (eg demographic, clinical, social) and information on exposures and potential confounders	5; Methods, Study design and setting	"The data was collected as a part of a retrospective cohort study over 13-year period at Chiang Mai University Hospital (CMUH) [8], a 1400-bed tertiary teaching hospital. The hospital is the largest in northern, Thailand and the fourth largest hospital in the country where most of patients in northern region from 17 provinces are referred to for tertiary care (see Table S1 List of Northern provinces of Thailand as of 2018). Cultural eating habit of raw pork dishes and fermented raw pork is commonly practiced in northern, Thailand. This deep-rooted cultural eating behavior is a major route of the disease transmission and contributing factor of a high prevalence of the disease in this region."
		(b) Indicate number of participants with missing data for each variable of interest	17; Table 2 Clinical characteristics of *S. suis* infected patients for hearing loss	"† Data available in 101 patients; ‡ Data available in 57 patients; ‡‡ Data available in 53 patients; †† Data available in 52 patients; ± Data available in 127 patients"
		(c) *Cohort study*—Summarise follow-up time (eg, average and total amount)	8; Results	"All SNHL still persisted based on audiometry upon discharge and

				latest follow-up visit up to December 2018.”
Outcome data	15*	*Cohort study*—Report numbers of outcome events or summary measures over time	8; Results	“More than one-third of patients had a history of raw pork consumption and nearly half of the patients were regular alcohol drinkers. Valvular heart disease was the most common underlying disease followed by Diabetes Mellitus (DM) and spondylodiscites. A total of 42 patients (31.58%) experienced SNHL from S.suis infection in which 13 (9.77%) were mild SNHL, 7 (5.26%) were moderate SNHL, 4 (3.01%) were severe SNHL and 12 (9.02%) were profound SNHL. All SNHL still persisted based on audiometry upon discharge and latest follow-up visit up to December 2018.”
		Case-control study—Report numbers in each exposure category, or summary measures of exposure	N/A	
		Cross-sectional study—Report numbers of outcome events or summary measures	N/A	
Main results	16	(*a*) Give unadjusted estimates and, if applicable, confounder-adjusted estimates and their precision (eg, 95% confidence interval). Make clear which confounders were adjusted for and why they were included	N/A	
		(*b*) Report category boundaries when continuous variables were categorized	9; Results	“Continuous variables were categorized into two groups to simplify application. For creatinine, the cut point was 2.0 mg/dL which was around 1.5 to 2

			times compared to the mean value among S.suis hearing loss cases whereas 4.0 mmol/L was the cut-off point for serum potassium.”
	(*c*) If relevant, consider translating estimates of relative risk into absolute risk for a meaningful time period	N/A	

Continued on next page

Other analyses	17	Report other analyses done—eg analyses of subgroups and interactions, and sensitivity analyses	9; Results<br><br>Supporting information; Table S2	"GCS variable remained a non-significant predictor in both complete case analysis and multiple imputation data (Table S2 Clinical characteristics of S. suis infected patients for hearing loss based on imputed GCS)."
**Discussion**				
Key results	18	Summarise key results with reference to study objectives	10;Discussion	"To our best knowledge, this is the first risk scoring system development for *S.suis* hearing loss. We identified meningitis, raw pork consumption and vertigo as the main risk factors of *S.suis* hearing loss. The data was derived from the real setting in routine practice upon admission at Chiang Mai University Hospital (CMUH). Usually these data are available and do not require any invasive laboratory procedure. After evaluating its external validity, this simple scoring system might be useful to assess patients with *S.suis* hearing loss in hospital and primary care settings."
Limitations	19	Discuss limitations of the study, taking into account sources of potential bias or imprecision. Discuss both direction and magnitude of any potential bias	11;Discussion	"However, a number of limitations can be noted in our study. Missing data and recall bias may have arisen due to retrospective nature……"

Interpretation	20	Give a cautious overall interpretation of results considering objectives, limitations, multiplicity of analyses, results from similar studies, and other relevant evidence	12-13; Discussion	"In conclusion, after external validation, our simple clinical risk score developed might be useful to aid clinicians in identifying patients who are likely to develop hearing loss from *S.suis* infection. Although this tool cannot replace clinical judgement, physicians can look upon these clinical characteristics in patients (meningitis, raw pork consumption and vertigo) for early detection of potential *S.suis* hearing loss cases and administrate immediate treatment to avoid long-term complications."
Generalisability	21	Discuss the generalisability (external validity) of the study results	12: Discussion	"Nevertheless, as traditional culture involving raw pork consumption is a well-known risk behavior among northern Thai population, doctors might have more likely asked patients about this risk behavior which might have been potentially subject to information bias. Therefore, generalizability of the finding should be done with caution."<br><br>"Finally, it should be noted that the participants included in the study were mainly from Northern, Thailand where traditional raw pork

				eating is practiced. This may limit generalizability in other settings especially where raw pork consumption is uncommon."
**Other information**				
Funding	22	Give the source of funding and the role of the funders for the present study and, if applicable, for the original study on which the present article is based	N/A	

*Give information separately for cases and controls in case-control studies and, if applicable, for exposed and unexposed groups in cohort and cross-sectional studies.

Note: An Explanation and Elaboration article discusses each checklist item and gives methodological background and published examples of transparent reporting. The STROBE checklist is best used in conjunction with this article (freely available on the Web sites of PLoS Medicine at http://www.plosmedicine.org/, Annals of Internal Medicine at http://www.annals.org/, and Epidemiology at http://www.epidem.com/). Information on the STROBE Initiative is available at www.strobe-statement.org.
