# Supplementary material for: A risk scoring system for predicting Streptococcus suis hearing loss: A 13-year retrospective cohort study
Source: PLoS One. 2020 Feb 4;15(2):e0228488. doi: 10.1371/journal.pone.0228488 (PMC6999904; doi:10.1371/journal.pone.0228488)
Supplement: S1 Table — (DOCX) [file pone.0228488.s003.docx]

**S1 Table. List of Northern provinces of Thailand as of 2018** (National Statistics Office (NSO). [cited 2019 22 July]; Available from: http://www.nso.go.th/sites/2014/nsopublic)

| **Upper Northern Thailand** | **Lower Northern Thailand** |
| --- | --- |
| Chiang Rai | Tak |
| Chiang Mai | Kamphaeng Phet |
| Nan | Nakhon Sawan |
| Phayao | Phetchabun |
| Phrae | Phichit |
| Mae Hong Son | Phitsanulok |
| Lumphun | Sukhothai |
| Lampang | Uthai Thani |
| Uttaradit |  |
