# Supplementary material for: A risk scoring system for predicting Streptococcus suis hearing loss: A 13-year retrospective cohort study
Source: PLoS One. 2020 Feb 4;15(2):e0228488. doi: 10.1371/journal.pone.0228488 (PMC6999904; doi:10.1371/journal.pone.0228488)
Supplement: S2 Table — (DOCX) [file pone.0228488.s004.docx]

**S2 Table. Clinical characteristics of *S. suis* infected patients for hearing loss based on imputed GCS**

| Characteristics | Hearing loss (n=42) | Non-hearing loss (n=91) | p-value |
| --- | --- | --- | --- |
|  | N (%) | N (%) |  |
| Demographics: |  |  |  |
| - Age (year) (mean±SD) | 54.62±12.12 | 57.33±14.33 | 0.290 |
| - Male | 29 (69.05) | 63 (69.23) | 0.568 |
| - Raw pork consumption | 25 (59.05) | 24 (26.37) | <0.001 |
| - Alcohol drinking | 25 (59.52) | 41 (45.05) | 0.138 |
| Baseline characteristics: |  |  |  |
| GCS † (Hearing loss n=41 ) | 13.39±0.37 | 12.76±0.34 | 0.268 |
| Microbiological results: |  |  |  |
| - Time to microbiological cure | 9.86±11.07 | 8.33±15.29 | 0.526 |
| - Mean MIC to penicillin (mcg/mL) ‡ | 0.14±0.18 | 0.17±0.15 | 0.106 |
| - Mean MIC to ceftriaxone (mcg/mL) ‡‡ | 0.14±0.10 | 0.29±0.31 | 0.388 |
| Underlying disease |  |  |  |
| - Valvular heart disease | 7 (16.67) | 37 (40.66) | 0.009 |
| - ALD | 2 (4.76) | 14 (15.38) | 0.093 |
| - DM | 5 (11.90) | 21 (23.08) | 0.162 |
| - Spondylodiscites | 6 (14.29) | 21 (23.08) | 0.354 |
| Major clinical manifestations |  |  |  |
| - Acute meningitis | 34 (80.95) | 16 (17.58) | <0.001 |
| - Neck stiffness | 31 (73.81) | 16 (17.58) | <0.001 |
| - Septicaemia | 23 (54.76) | 51 (56.04) | 1.000 |
| - IE | 4 (9.52) | 30 (32.97) | 0.005 |
| - Vomiting | 14 (33.33) | 13 (14.29) | 0.019 |
| - Vertigo | 7 (16.67) | 3 (3.30) | 0.011 |
| Receiving steroids | 22 (53.66) | 1 (1.10) | 0.600 |
| Laboratory findings: |  |  |  |
| - CSF protein†† | 277.12±220.25 | 341.39±256.93 | 0.419 |
| - CSF glucose‡‡ | 31.69±9.70 | 30.72±21.55 | 0.173 |
| - Creatinine (mg/dl) | 1.18±0.75 | 2.09±2.94 | 0.018 |
| - Potassium (mmol/L)± | 3.52±0.43 | 3.93±0.77 | 0.002 |

ALD, Alcoholic liver disease; CSF, Cerebrospinal fluid; DM, Diabetes Mellitus; GCS, Glasgow coma scale; IE, Infective endocarditis; MIC, Minimal Inhibitory Concentration

† Data was imputed in 31/32 missing GCS values due to there was one missing parameter to estimate GCS in 1 patient.
