# Supplementary material for: A risk scoring system for predicting Streptococcus suis hearing loss: A 13-year retrospective cohort study
Source: PLoS One. 2020 Feb 4;15(2):e0228488. doi: 10.1371/journal.pone.0228488 (PMC6999904; doi:10.1371/journal.pone.0228488)
Supplement: S3 Table — (DOCX) [file pone.0228488.s005.docx]

**S3 Table. Hosmer-Lemeshow good-ness-of-fit test**

| **Group** | **Probabilities** | **Obs_1** | **Exp_1** | **Obs_0** | **Exp_0** | **Total** |
| --- | --- | --- | --- | --- | --- | --- |
| 4 | 0.0571 | 5 | 3.3 | 52 | 53.7 | 57 |
| 6 | 0.1759 | 3 | 4.0 | 20 | 19.0 | 23 |
| 7 | 0.5018 | 9 | 11.2 | 15 | 12.8 | 24 |
| 9 | 0.7803 | 18 | 17.2 | 4 | 4.8 | 22 |
| 10 | 0.9473 | 7 | 6.3 | 0 | 0.7 | 7 |

Number of observations = 133

Number of groups = 5

Hosmer-Lemeshow chi2(3) = 3.13

Prob > chi2 = 0.3721
