# Supplementary figures and images for: A risk scoring system for predicting Streptococcus suis hearing loss: A 13-year retrospective cohort study
Source: PLoS One. 2020 Feb 4;15(2):e0228488. doi: 10.1371/journal.pone.0228488 (PMC6999904; doi:10.1371/journal.pone.0228488)

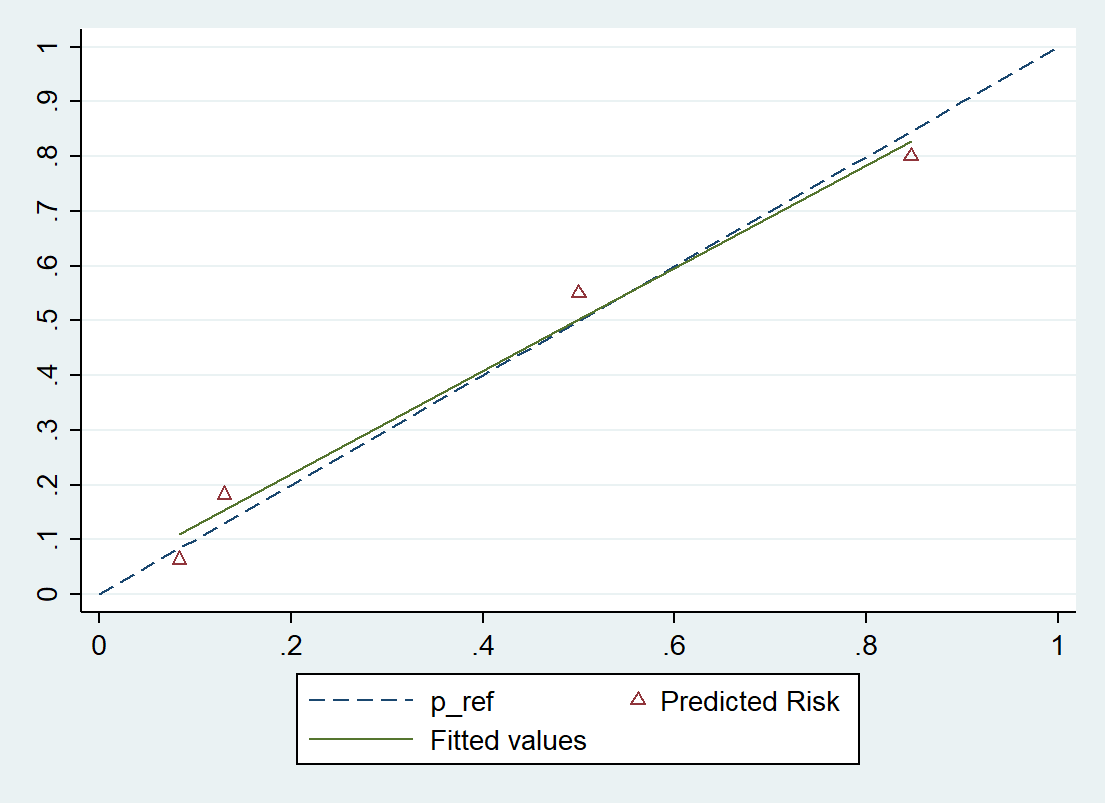

Supplement: S1 Fig — (TIF) [file pone.0228488.s006.tif]

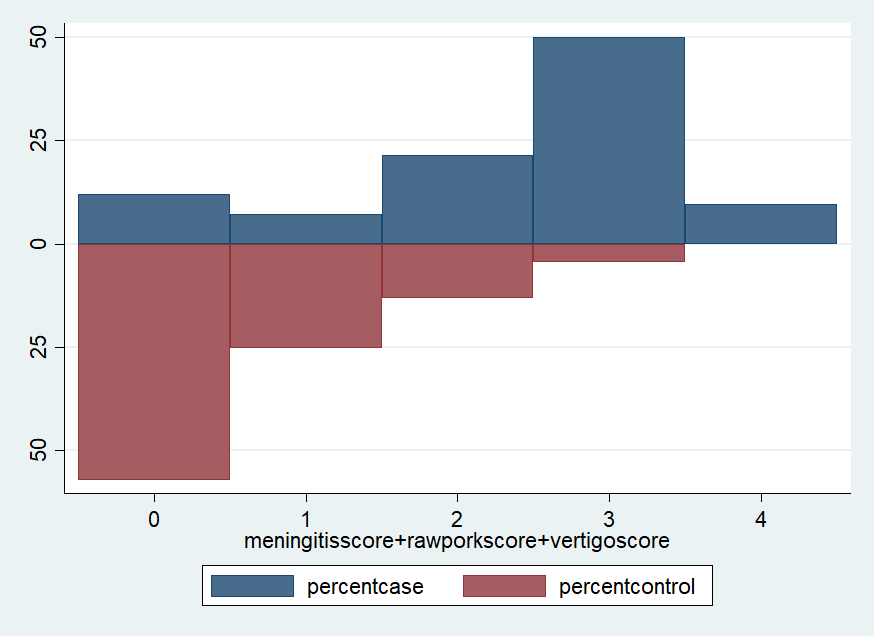

Supplement: S2 Fig — (TIF) [file pone.0228488.s007.tif]
